# Exploring the Reasons for Low Cataract Surgery Uptake Among Patients Detected in a Community Outreach Program in Cameroon: Focused Ethnographic Mixed Methods Study

**DOI:** 10.2196/35044

**Published:** 2022-06-09

**Authors:** Mathew Mbwogge, Henry Ebong Nkumbe

**Affiliations:** 1 See Acknowledgments London United Kingdom; 2 Africa Eye Foundation Cameroon Yaoundé Cameroon

**Keywords:** ophthalmologic surgical procedures, access to health care, ophthalmology, patient-centered care, ethnography, health knowledge, attitudes, practice

## Abstract

**Background:**

Vision 2020: The Right to Sight, was one potential way to deal with the barriers surrounding cataract surgery and improve access to eye care. To this effect, the Magrabi International Council of Ophthalmology (ICO) Cameroon Eye Institute (MICEI) has performed more than 1000 sight-restoring cataract surgeries among patients referred from outreach camps. However, quite a good number of patients diagnosed with cataracts during community screening camps fail to present for surgery. This study sought to explore some of the challenges to accepting cataract surgery among community-diagnosed patients with cataract, patients operated for cataract, and community members.

**Objective:**

The study objective was 5-fold: (1) to assess the level of awareness about cataract and available treatment, (2) to explore barriers to cataract surgery uptake, (3) to assess people’s perception about the outcome of cataract surgery, (4) to understand people’s perception about free cataract surgery, and (5) to explore reasons for outright refusal of cataract surgery.

**Methods:**

This was a focused ethnographic study from December 2018 through February 2019 in 3 different communities of the Center Region of Cameroon, in which patients with cataract were diagnosed. The study sample was composed of patients operated for cataract, those diagnosed with cataract, key informants, and community members. Focus group discussions (FGDs), personalized in-depth interviews, and a short demographic questionnaire were used to collect data. Data were analyzed using a Microsoft Excel spreadsheet and Stata 14 (StataCorp). Data were presented using tabular and graphical methods.

**Results:**

A total of 29 subjects (19 men) with a mean age of 54.5 (SD 14.5) years took part in the study. The most prominent barriers to cataract surgery were found to be cost (25/29, 86%) and fear of surgery (17/29, 59%). It was also noted by 41% (12/29) of subjects that those who do not take up cataract surgery turn to traditional medicine. Other barriers included the lack of awareness of available treatment (6/29, 21%), no perceived need (5/29, 17%), cultural beliefs and superstition (4/29, 14%), and negligence (4/29, 14%).

**Conclusions:**

We found cost (25/29, 86%) and fear (17/29, 59%) to be the main barriers. Belief in traditional medicine and superstition were the main drivers of fear. The implementation of a tiered pricing system, counseling training for key informants, incentives for the referral of patients with cataract, mass media engagement, advocacy, training and active involvement of traditional doctors as key informants, acquisition of a 4×4 outreach van, and motorbikes for camp organizers were some of the recommendations based on our results.

## Introduction

**Figure 1:**
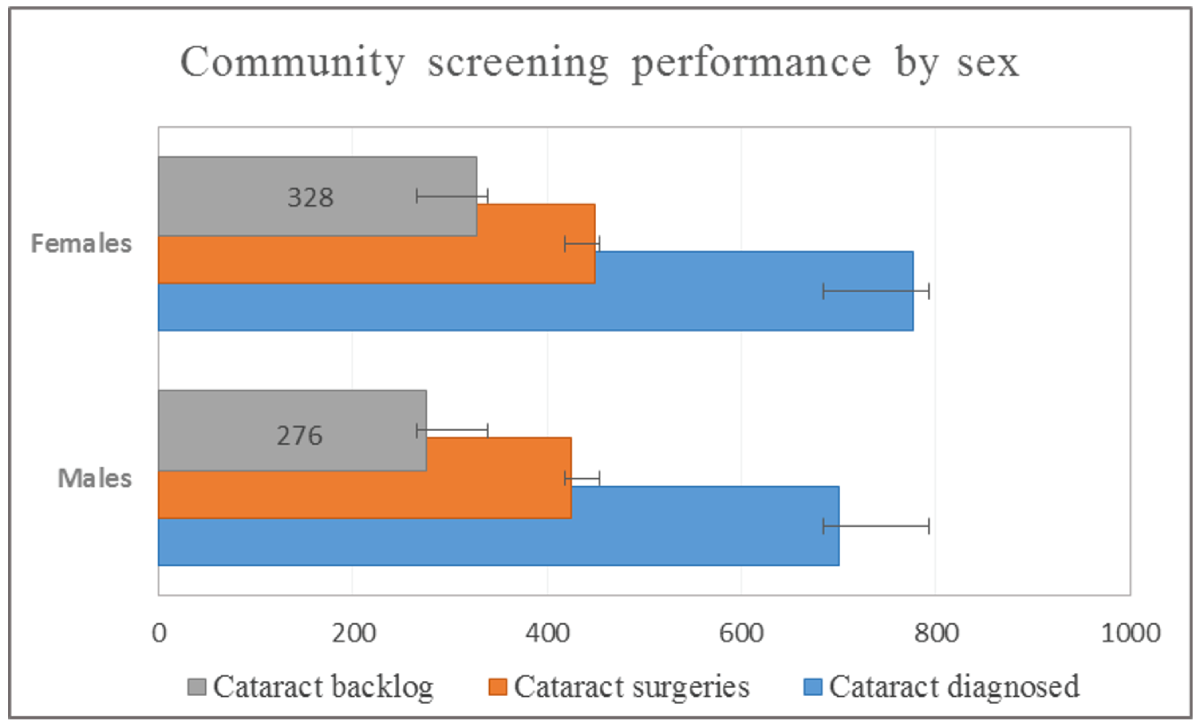
Community-diagnosed cataract backlog in 2018.

According to VISION 2020: The Right to Sight, African countries needed a cataract surgical rate of at least 2000 per million population to eliminate avoidable blindness by 2020 [1,2]. The Magrabi International Council of Ophthalmology (ICO) Cameroon Eye Institute (MICEI) [3], in attempting to expand high-quality and subsidized cataract surgeries through free community eye screening camps, saw a backlog of 40.9% (604/1477) of diagnosed cataracts. Figure 1 shows the backlog of community-diagnosed cataracts for 2018.

The global burden of blindness increased by 10.8% between 2010 and 2019 [4]. This burden was worse in sub-Saharan Africa owing to limited eye care personnel [5,6]. Studies have shown that the prevalence of moderate to severe bilateral visual impairment among those aged 50 years and above is approximately 10.9% (95% CI 8.3-14.3) [7]. Cataracts are considered the leading cause of blindness among the ≥50-year age group, and 55% of those blind individuals are women [8]. Although approximately 80% of blindness is preventable with either a simple sight-restoring surgery or a pair of eyeglasses, many people, particularly older individuals in the community, continue to be needlessly blind [9]. The Universal Eye Health Global Action Plan (UEH GAP 2014-2019) was aimed at a world in which no one is needlessly blind and those with irreversible blindness can achieve their full potential by integrating eye health into national health plans [4,10-12]. The fact remains that low- and middle-income countries are disproportionately affected with 90% of the global burden borne by African countries and with vulnerable low-income individuals particularly being the hardest hit [13].

The Universal Health Coverage effectiveness coverage index for Cameroon is 42 [14] and 70% of the total health spending per capita (US $60, range, US $47-75) is an out-of-pocket expense [15]. This may explain why Cameroon has one of the highest burdens of moderate to severe visual impairment in the world [16], with almost a quarter of a million persons reported to be blind and approximately 720,000 individuals with visual impairment [16]. Age remains a major predictor and as such, the burden of visual impairment in Cameroon increases with age [17]. There is limited evidence regarding the health-seeking behavior of different communities toward eye care in Cameroon. Studies conducted (most of which were hospital-based and dating more than a decade) have focused on visual impairment, causes, and functional difficulties [18-22]. We found a single community-based study related to self-reported visual impairment [9]. There is the staggering belief in Cameroon that those with health concerns report to the hospital, but evidence suggests that Cameroonians generally report to health facilities when their health conditions have worsened [23,24].

Despite having a National Eye Care Program (Programme national de lutte contre la cécité), there is currently a very small government budget compared to needs specifically allocated to eye care as reported in many other sub-Saharan countries [25]. Lack of integration of eye care into the public health strategy, unavailability of well-trained personnel, and concentration of those trained in major city centers [26,27] coupled with inappropriate infrastructure limit access to eye care among high-income individuals. This is further compounded by the poor transport network, ignorance of available services, and cultural beliefs [28]. This limits the cataract surgical rate of Cameroon—which is the number of cataract operations performed per million population per year—to 758, which is far below the recommended 2000 (target) [29]. Eye care delivery in Cameroon is hospital-based and the evidence on best practices, for which particular groups of persons they are intended, and under what contextual factors they are delivered is generally lacking [30]. The above-highlighted challenges leave the prevalence of cataract blindness in the community very high among the elderly population, thereby making traditional medicine an important alternative [31].

**Figure 2:**
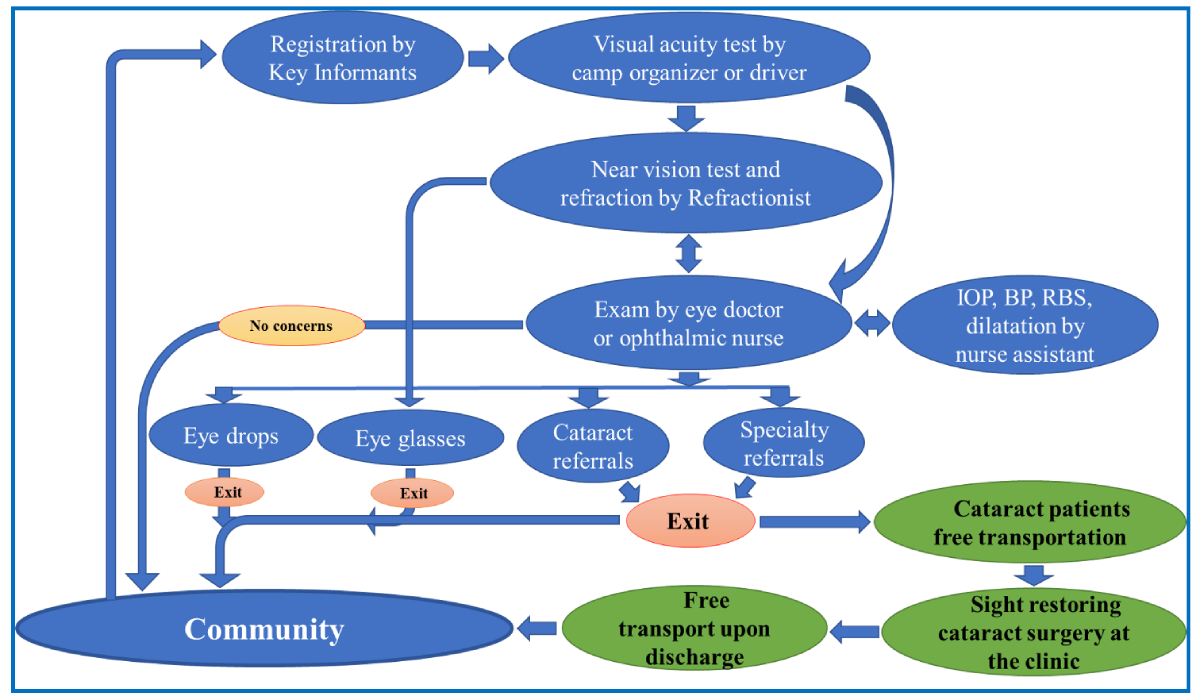
Magrabi International Council of Ophthalmology Cameroon Eye Institute’s community ophthalmic screening camp workflow. BP: blood pressure, IOP: intraocular pressure, RBS: random blood sugar.

The establishment of MICEI led to the introduction of the Aravind eye care delivery model [32-34] in Cameroon. This program involves community screening targeted to the ≥50-year age group, providing free voluntary transport referral to the clinic and back to the community for those diagnosed with cataract, offering sight-restoring cataract surgery, a bed, feeding, and postoperative medications. Figure 2 shows the community screening camp’s workflow.

A similar community-based tiered system is operational in Nigeria [35,36] either by the government or private clinics, Ethiopia [37], and in the Democratic Republic of the Congo [38]. Patients admitted for surgery may be operated on site, as is the case in Nigeria. Community screening programs specific to cataract surgery may also take the form of operationalizing memoranda of understanding with donor organizations. This includes, for example, the Seeing Is Believing project (SiB) [39], the Hilton Cataract Initiative [40], and the Cameroon Cataract Bond [41,42].

We aimed at understanding the challenges associated with accessing cataract surgery. The study objectives were to (1) assess the level of awareness about cataracts and available treatment, (2) explore barriers to cataract surgery, (3) assess the community’s perception about the outcome of cataract surgery, (4) understand people’s perception surrounding free cataract surgery, and (5) understand the reasons for outright refusal of cataract surgery.

## Methods

### Study Design

This was an ethnographically oriented mixed methods study [43-45] involving informal discussions, outreach document analysis, field visits, field notes, focus group discussions (FGDs), in-depth interviews, and questionnaires. We focused the ethnography [46] on investigating the challenges surrounding decisions to take up cataract surgery.

### Ethnographic Rationale

Ethnography [47,48] in health care is a context-specific and field-based approach to understanding patient behavior within the complexity of their family and community cultures [49,50]. Ethnography is a recommended approach for multisite studies aimed at capturing diverse perspectives [51]. Evidence also suggests that ethnography can be focused [52,53] to address specific issues or questions. This approach was particularly important because we wanted to understand not only the reasons for the low uptake of cataract surgery among patients with cataract but also the role of family members and the community as a whole in the making of such decisions [46,54]. Studies have used similar methods in exploring user experiences in health care [55,56].

### Research Team and Reflexivity

The research team comprised (1) the principal investigator (PI), (2) a research assistant recruited purposely for the study, (3) a trained key informant, and (4) a driver. While subjects were known to the research key informant, a few might have seen the driver during screening camps. The PI, who was familiar with qualitative research methods, drilled the research assistant and the driver on the study and data collection procedures prior to the study. The key informant was briefed about the study over the telephone but only knew about the questions during FGDs. Both the PI and the driver administered open-ended questions during FGDs while the PI took on the personalized interviews. The key informant assisted with translation during FGDs and only assisted in personalized interviews on the basis of need.

### Setting and Context

This study took place in 3 underprivileged communities in which MICEI has organized outreach screening campaigns. MICEI is a 73-bed capacity lone subspecialty clinic and training institute dedicated to eye care in Cameroon and environs, with a daily traffic of 300 outpatient visits [3]. The clinic is home to 7 ophthalmologists, 7 ophthalmic nurses, an optometrist, a low vision expert, 75 allied eye health personnel, and 2 cataract operating rooms [57].

The 3 ethnographic study sites were predominantly French-speaking communities located in the Lekié Division of the Center Region of Cameroon including Elig-Mfomo in the Elig-Mfomo Sub-Division, Nkalngaha in the Evodoula Sub-Division and Lenouk in the Monatélé Sub-Division. The Lekié Division is inhabited by half a million population dispersed across 700 villages. Life is subsistent and highly reliant on agriculture, with cocoa as the main cash crop [58].

The population of the Center Region of Cameroon is approximately 4.5 million. There are 8 other eye clinics (general ophthalmology) within the region including the (1) University Teaching Hospital, (2) Central Hospital, (3) General Hospital, (4) Military Hospital, (5) Gyneco-obstetric Hospital, (6) Etoug-Ebé Presbyterian Hospital, (7) Essos Hospital Center-NSIF, and the (8) Obala Sub-Divisional Hospital.

### Sampling Strategy

#### Study Site Selection

Ahead of the study, an assessment of the community outreach program showed a backlog of 42.13% (495/1175) of all diagnosed cataracts. We compared the number of people with cataract diagnosed in each community visited in 2018 to the number of those who received surgery. Since the aim was to improve the cataract acceptance rate through the emic and etic perspectives [59], a study exercise of outreach reports together with informal conversations with colleagues provided insights on the communities with extremely poor and best cataract surgery acceptance rates.

#### Recruitment of Participants

A total of 29 subjects were recruited from a sampling frame of both operated and unoperated patients with cataract as well as the members of each selected community. For good representation, each community sample constituted 3 direct subjects (1 operated and 2 unoperated patients with cataract) and 7 indirect subjects (1 family head or breadwinner of the operated patient, 1 family head or breadwinner of an unoperated patient with cataract, a village head, a traditional healer, a trained key informant, a trained frontline health worker, and an influential community member). Both the operated and the unoperated patients with cataract were purposefully selected from the clinic’s database of operated and diagnosed patients with cataract followed by a snowball sampling technique, with the help of key informants. A purposive sampling technique was used to identify key informants from the sampling frame of the MICEI’s trained key informants from the preselected study sites. The inclusion criteria were (1) being diagnosed in a screening camp organized by the clinic, (2) being diagnosed in the selected community, (3) being a resident within the community and environs, and (4) having undergone surgery at the MICEI. The exclusion criteria were (1) site inaccessibility, (2) inability to provide informed consent, and (3) inability to communicate.

### Ethical Considerations

A study protocol was developed and informally approved by the institutional review board (IRB) of the MICEI. Written informed consent was sought from all participants in accordance with the tenets of the Helsinki Declaration of 1975 [60]. This study adhered to the Critical Appraisal Skills Program [61,62]. Subjects were briefed prior to data collection, on the need to maintain anonymity. Research materials were translated into the French language as the study sites were predominantly French-speaking communities. Participants received reimbursement for their transport fares. All blind subjects with cataract were invited for free cataract surgery. All questions were translated into the local Eton or Manguissa languages (subsets of the Beti ethnic group) for those who neither understood nor could express themselves in the official languages. All the concerns of subjects were addressed before the start of data collection.

### Data Collection Procedure

An interview guide (Multimedia Appendix 1) and a short demographic questionnaire were developed and reviewed by the IRB in a research protocol ahead of field visits. We consulted with colleagues to define the questions that preoccupied the community eye health unit, and then we used a realist approach [63] to develop the interview guide based on a framework of the defined questions as main themes. Interview guide questions included “What do you know about cataract?” “What would you say hinders people from taking cataract surgery?” “What do people say when those who had cataract surgery return to the community?” “What is your opinion about free and paid cataract surgery?” and “Why do you think some people do not want cataract surgery?” All data were collected in the French language, and translations were made into local languages and vice versa. Data collection was organized into 3 FGDs (N=30), 30 personalized interviews, and 30 short sociodemographic questionnaires. Data were collected on December 28, 2018, for Nkalngaha, January 3, 2019, for Elig-Mfomo, and February 7, 2019, for Lenouk. One participant was absent, thereby reducing the sample to 29 subjects.

#### Document Review

Data on which sites were to be selected were obtained through document analysis of the community outreach unit at the base clinic. It took the form of a desk exercise followed by a deliberation meeting between the PI and colleagues. The review was based on outreach, operating theater, and medical record reports from January through September 2018. Data from the reports generated by the 3 departments were matched for consistency. We then calculated the cataract backlog by comparing the number of operated patients with diagnosed ones. This was used to produce time-series graphs per outreach site in Microsoft Excel.

#### FGDs

All subjects took part in a community-based FGD at each of the 3 study sites. Focus groups were a cross-section of the communities from which they were drawn. The duration of FGDs, which took place at a venue arranged by participants, ranged 30-42 minutes. FGDs were conducted by both the driver and the PI using open-ended questions. Participants took turns to express their views and experiences without time restrictions. The PI moderated unnecessarily lengthy discussions and used probes until saturation was reached. Saturation was observed when there was silence with no further contributions. The key informant switched between the moderate role (by translating into the local language and vice versa) and an “observer as a participant” role during FGDs [64].

#### Personalized Interviews

All participants were invited to a personalized interview with the PI at the end of each FGD. Interviews were conducted further away from FGDs to enhance autonomy. The key informant assisted with translation where necessary. The overall duration of personalized interviews was 66 minutes on average.

#### Demographic Questionnaire

The data on demographic variables were also collected by the research assistant at the end of personalized interviews. All 29 subjects took part in the survey. The place for administering the short questionnaire was decided at the convenience of participants.

#### Field Backup Notes

The research assistant who served as a complete observer (passive participant) took backup notes during FGDs on the basis of observed and nonverbal communication of subjects as well as any phenomenon of interest, using eye-to-eye and soul-to-soul approaches.

### Data Collection Tools

An interview guide was used to conduct the FGDs and personalized interviews. Digital recordings of FGDs and interviews were performed using an Android tablet (SAMSUNG Galaxy Note 10.1). A short paper questionnaire was used to capture data on age, sex, marital status, residence, education, and occupation. Field and backup notes were collected on A4 papers. The distances of participants' villages from the clinic were computed using Google and OpenStreetMap [65].

### Data Processing and Analysis

#### Data Preparation

Digital recordings of FGDs and personalized interviews were assessed after each field visit for quality and saved against a date and study site. Captured demographic data were entered into a Microsoft Excel spreadsheet prior to analysis. Audio recordings were severally listened to vertically and horizontally for familiarization. Based on this intimacy, the data were transcribed and translated into the English language. For the easy discovery of phrases of interest in the data, investigation, and analysis, we adopted a heuristic coding approach [66] starting with questions, including “What do you know about cataract?” (Code 1), “What would you say hinders people who want surgery from taking cataract surgery?” (Code 2), “What do people say when those who had cataract surgery return to the community?” (Code 3), “What is your opinion about free and paid cataract surgery?” (Code 4), and “Why do you think some people do not want cataract surgery?” (Code 5).

#### Data Analysis

##### Quantitative Analysis

The quantitative data analysis of this study started with the outreach unit review process whereby we compared the monthly reports of community screening camps. This was trimmed down to the 3 sites with the poorest performances by comparing the monthly cataract diagnosed lists established at the end of each camp against the monthly operating theater reports of those who received surgery, for the period of January to September 2018. Demographic survey data were analyzed using Stata 14 (StataCorp).

##### Thematic Analysis

The inductive and deductive methods, known to be effective in exploring users’ views [67], were used to analyze qualitative data. An inductive approach and predefined framework [68] were used to focus the thematic analysis on research objectives and interview guide [69]. FGD and interview data were transcribed and analyzed thematically [70] using Microsoft Excel. Through a heuristic process [66,71], multiple rounds of going through the transcribed data and field notes permitted the identification of phrases linked to the originally established question codes. These were then arranged into themes in accordance with question codes by means of cut and paste. Further investigation into these themes (deduction) led to the breakdown of these themes into subthemes, which provided insights into the different pieces of information required for each question. These pieces were analyzed by focusing on content and context with the intention of creating new knowledge about individual perceptions, how they are interrelated, and their relation to the environment [66]. Each subtheme (coded datum) was attributed a descriptive code [72] that depicted the datum’s intent and essence.

##### Data Transformation

In this study, contextualized qualitative data were transformed by giving numerical meaning to quotes (scoring) [73] on the basis of “popularity coding” for robust presentation and visualization [74]. Although some have criticized the quantification of qualitative data [75], our proposed theme and subtheme “popularity coding” approach is based on the argument that the finality of data analysis is to meaningfully represent data and arrive at conclusions that mirror the data [76]. Besides, the coding in thematic analysis, as well as logistic regression techniques, still represent the use of numerical values in qualitative data. We depicted the relative importance attached to particular words and experiences based on how often they appeared in the text. These were then assigned numerical values and reported as frequencies.

### Trustworthiness, Validity, and Reliability

The use of focused ethnography [52,53] led to the diversity of captured data and rendered it as close to reality and specific to communities as possible. Prompts proposed by Dixon-Woods et al [77] were used to ensure quality. Trustworthiness in the results was ensured by establishing a link and maintaining harmony between the data and the analysis through a back-and-forth approach, by continually listening to the audios and reading the transcribed data intermittently to ensure coherence, intimacy, incubation, and reflexivity [66]. The triangulation of data from FGDs and personalized interviews from 3 different communities increased the internal validity of our results. Intermittently withdrawing from the data analysis and enthusiastically returning to the transcriptions led to inspirational fresh immersion, incubation, and reflexivity, which improved the way data were analyzed and interpreted [78]. Notwithstanding the challenges of measuring reliability in qualitative research [79], specific reporting guidelines were used to maintain reliability and ensure that the results are reproducible.

## Results

### Results Overview

We report the study findings using the Critical Appraisal Skills Program [61,62], the Standards for Reporting Qualitative Research [80], as well as the recommendations of Gertner et al [51] for reporting studies with ethnographic approaches.

### Participant Characteristics

Table 1 shows the demographic characteristics of participants. Altogether 29 subjects from 3 different communities (Evodoula, Elig-Mfomo, and Monatélé Sub-Divisions) were recruited to the study from December 2018 through February 2019. The age of the subjects ranged 30-81 years with a mean age of 54.5 (SD 14.5) years. Male subjects constituted 66% (19/29) of the total sample. Subjects came from 7 different villages with an average distance of 82.5 km and a drive time of 2 hours 23 minutes from the eye clinic (Table 2).

**Table 1:** Participant demographic characteristics (N=29).

Category			Direct (n=9), n (%)		Indirect (n=20), n (%)		Total, n (%)
**Focus group**							
	Focus group discussion 1	3 (33)		6 (30)		9 (31)	
	Focus group discussion 2	3 (33)		**7** (35)		10 (35)	
	Focus group discussion 3	3 (33)		7 (35)		10 (35)	
**Age (years)**							
	<40	—^a^		3 (15)		3 (10)	
	40-49	2 (22)		10 (50)		12 (41)	
	50-59	3 (33)		2 (10)		5 (17)	
	60-69	2 (22)		1 (5)		3 (10)	
	70-79	1 (11)		2 (10)		3 (10)	
	≥80	1 (11)		2 (10)		3 (10)	
**Reported sex**							
	Male	5 (55)		14 (70)		19 (66)	
	Female	4 (44)		6 (30)		10 (35)	
**Marital status**							
	Married	6 (67)		13 (65)		19 (66)	
	Cohabiting	1 (11)		4 (20)		5 (17)	
	Single	—		—		—	
	Divorced/widowed	2 (22)		3 (15)		5 (17)	
**Residence**							
	Nkalngaha (Evodoula)	3 (33)		6 (30)		9 (31)	
	Elig-Mfomo	3 (33)		7 (35)		10 (35)	
	Lenouk (Monatélé)	2 (22)		4 (20)		6 (21)	
	Monatélé urban	—		1 (5)		1 (3)	
	Akougouda (Monatélé)	1 (11)		—		1 (3)	
	Nkol-Evida (Monatélé)	—		1 (5)		1 (3)	
	Nkolngal (Monatélé)	—		1 (5)		1 (3)	
**Employment**							
	Yes	—		3 (15)		3 (10)	
	No	9 (100)		17 (85)		26 (90)	
**Education**							
	None	1 (11)		3 (15)		4 (14)	
	Primary	3 (33)		9 (45)		12 (41)	
	Ordinary secondary	4 (44)		5 (25)		9 (31)	
	Advanced secondary	1 (11)		2 (10)		3 (10)	
	Graduate	—		1 (5)		1 (3)	
	Postgraduate	—		—		—	

^a^—: not reported.

**Table 2:** Distance from the eye clinic.

Village	Distance (km)	Drive time (hours:minutes)
Average values	82.5	2:23
Nkalngaha (Evodoula)	44	1:15
Elig Mfomo	33	1:04
Lenouk (Monatélé)	100	2:52
Monatélé urban	91.2	2:36
Kougouda (Monatélé)	111.2	3:09
Nkolevida (Monatélé)	101.2	2:56
Nkolngal (Monatélé)	97	2:50

### Knowledge And Awareness About Cataract

**Figure 3:**
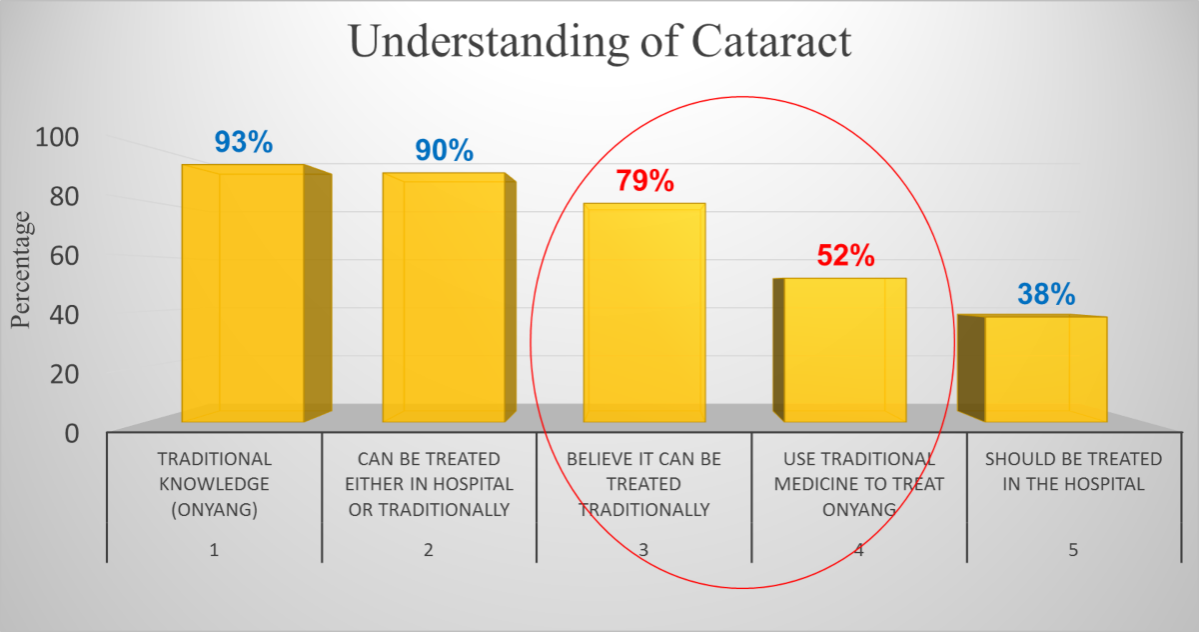
Participant-reported understanding of cataract.

Subjects perceived cataract as a disease that affects the eye and can be called by many names depending on the community. Up to 93% (27/29) of participants knew cataract either as “Onyang,” “Oquan-à-dis,” or “Ndem-à-dis.” According to them, “Onyang” was any visible white spot or substance in the eye that can either be treated traditionally or in the hospital. Further, sight loss to them meant that someone has the disease that affects the eye (cataract). Subject 2 in focus group 2 said, “Cataract is a disease that kills the eye, when it grows the eye dies.” Only 38% (11/29) mentioned the hospital as the appropriate place to seek treatment for cataracts, as can be seen in Figure 3.

The experience of Subject 6 attests to the fact that “Onyang” can be cured traditionally as indicated below:

It is something that develops in the iris and it is white. I can treat it but if it is more than me, I send the person to the hospital.

Indirect, Subject #6, FG1

### Perceived Barriers to Cataract Surgery

**Figure 4:**
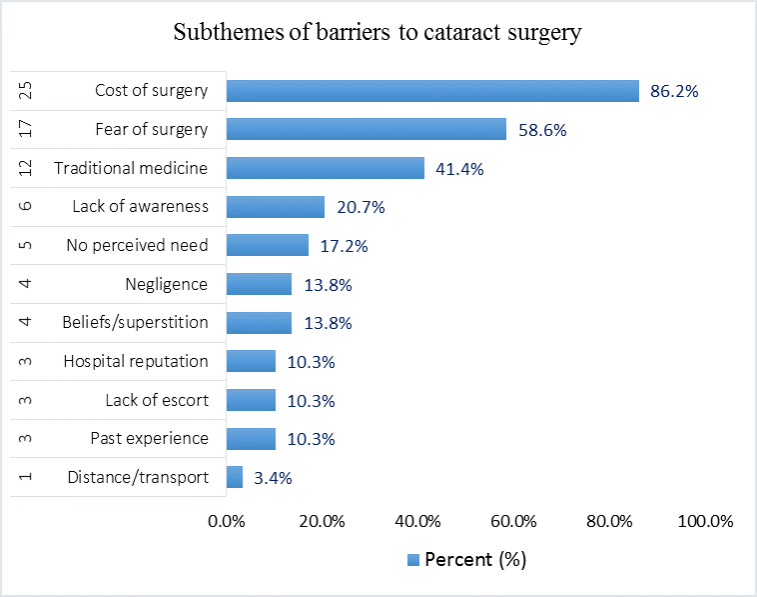
Subthemes that emerged as barriers to cataract surgery.

Barriers to cataract surgery, which emerged from FGDs common to all 3 communities included the cost of surgery, fear, and hospital reputation, particularly owing to a history of cataract surgeries with poor outcomes from other clinics. A comparison of the FGDs with personalized interviews showed that 86% (25/29) and 59% (17/29) of subjects noted that lack of money and fear of surgery were, respectively, the main barriers to their accessing cataract surgery. Further, 21% (6/29) of subjects also reported a lack of awareness of available treatment. Curiously, up to 41% (12/29) of subjects reported that those who fail to take up surgery turn to traditional medicine, which itself is a major barrier. Figure 4 shows the barriers that emerged from the transcripts.

The following excerpts demonstrate that people attend the hospital when their health situation has worsened:

…. It is Onyang in Etone [famous tribe]. When you have Onyang, they can use traditional medicine, it works with the Beti [famous ethnic group] but sometimes it fails, after that, you can go to the hospital.

Indirect, Subject #2, FG2

We used to go to herbalists […]. Like the ones of Papa, we tried to put traditional medicines, it did not work, we called, the first person came it did not work, the other one said ‘if I put the medicine two times it does not work then it is not at my level’.

Direct, Subject #2, FG3

In addition to traditional medicine as an alternative to treating cataracts in these communities, 28% (8/29) of interviewed subjects also reported that these patients stay at home, while only 13.8% (4/29) of subjects reported that patients sought care from other health facilities after failing to take up cataract surgery. One of the reasons why people sought traditional medicine was a lack of awareness of available treatment. One of the subjects said, “…we treat it with our leaves in the bush, herbs, we did not know that it can be operated” [FGD 3 extract].

### Perception About Operated Patients With Cataract

In general, positive feedback from the community when operated patients with cataract returned from the eye clinic was acknowledged across all 3 FGDs. This was also confirmed by most subjects during personalized interviews. It was reported that many are seen dancing when those operated for cataract returned to these communities, as shown in the following quotes:

They [community members] start doing propaganda, ‘such and such a person was at the hospital, he has been treated and now can see well’ […]. Many people congratulate the hospital staff […], they give positive comments.

Direct, Subject #3, FGD1

After your campaign, I saw a mother dancing at the center [health center] saying ‘I could not see but now I can see.’ those who did not go said ‘weeh!! [Exclamation], we have missed’.

Indirect Subject #6, FGD2

Approximately 86% (25/29) of interviewed subjects acknowledged that the community feedback is positive and that people are happy when those operated for cataract return into the community. Most of the patients reported that some are happy and others are not. The 5 main reasons that emerged from the data relating to why people might not be happy included lack of money to also undergo surgery (8/29, 28%), hatred and jealousy of those who were operated (4/29, 14%), regret (3/29, 10%), and the inability to continue managing the assets of those who were blind (7%). The following excerpts highlight some of the reasons:

Everybody says ‘it is good, people eat 3 times a day [at MICEI], there is a bed, dresses’. People complain because they do not have the XAF25,000 (US$42.86) for surgery.

Indirect, Subject #8, FG2

… what is sure is that the person who was blind and is now seeing is like a witch or wizard, as if he has done some magic, has taken the eyes of a sheep to use. People will start commenting that eyes have been purchased and given to you.

FGD3 extract

### Perception of Free Cataract Surgery

Subjects’ perception that people were positive about free cataract surgery dominated in 2 of the 3 FGDs. The discussion in one focus group weighed on the fact that free cataract surgery raises suspicion and fear as shown in the following extract:

It is suspected in the community because we know that what is free later becomes expensive. Also, we do not have confidence in such operations because there could be other motives.

Direct Subject #7, FG3

Members of some of the FGDs, however, believed that suspicion and fear should not be a major call for concern since surgery is aimed at helping people regain sight. In FGD 2, for instance, subjects reported that paid cataract surgery will only be appreciated by those who can afford to pay the requested contribution, while in FGD 3, members thought that people are generally more comfortable to pay when surgery is subsidized than when it is free. The main reason behind not being happy for paid cataract surgery was the lack of means as 41% (12/29) of subjects related their unhappiness to cost when they see those who underwent cataract surgery return into the community rejoicing.

In total, 79% (23/29) of subjects reported that they would be happy with free cataract surgery and that the community will be positive about it as well. One of the interviewees said, “I ask if true and I run for it” (Direct, Subject #1, FG1).

### Perceived Reasons for Refusing Cataract Surgery

In addition to participants’ lived experiences in accessing cataract surgery and their perception of free cataract surgery were other perceived challenges even if they were to be made free of charge. While during FGD 1, subjects thought that refusal of free cataract surgery could be attributed to ignorance, both FGD 2 and FGD 3 revealed that refusal may result from age, fear, cultural beliefs, and superstition. Concurrent with in-depth interviews, the most prominent reasons for outright refusal of cataract surgery were fear (9/29, 31%) and cultural beliefs and superstition (8/29, 28%). Other reasons included age (4/29, 14%), poor experience (3/29, 10%), ignorance (3/29, 10%), and postsurgical follow-up costs (2/29, 7%).

Some people traditionally cannot be operated, sometimes age, fear. Before they used to take people to [...], [an eye clinic: name removed], they will leave here seeing well, when they come back they are blind.

Indirect, Subject #8, male FGD2

… myself, I was operated on. […] but when I got home, my village brothers started quarreling with me saying ‘OK, we shall then see since you said you have gone to the hospital […]’. They are doing everything to put us uncomfortable [sorcerers], also do everything to save us [MICEI].

FG3 Discussion extract, Direct Subject

Regarding possible remedies to improve the uptake of cataract surgery, 24% (7/29) of subjects suggested the continuation of screening campaigns, 14% (4/29) suggested community education and awareness, and 14% (4/29) suggested counseling, including the use of postsurgical cataract ambassadors.

## Discussion

### Principal Findings

We found in this study that the barriers to cataract surgery included A (Awareness, Age), B (Beliefs or superstition, Bad experience), C (Cost), D (Distance), E (Escort), and F (Fear) among others. Even though 93% (27/29) of subjects knew cataract as “Onyang,” “Oquan-à-dis,” or “Ndem-à-dis” and as a disease that leads to blindness if not treated, up to 79% (23/29) of subjects believed cataract can be treated traditionally. In addition to seeking traditional treatment (12/29, 41%), cost (25/29, 86%) and fear of surgery (17/29, 59%) were the most acknowledged and leading barriers to cataract surgery. We also found that while most people were happy with free cataract surgery (23/29, 79%), cultural beliefs and superstition was a major driver of people’s fear of cataract surgery and lack of resilience.

### Interpretation of Results

We found in this study that 93% (27/29) of the study subjects knew about cataract as a potentially blinding eye disease, which was only a little lower than the 98% rate reported among 4 districts in Kerala, India [81]. Apart from a very large sample size of 2000, India has a long-standing history of community eye care delivery. Further, the 38% (11/29) of subjects who reported that cataracts should be treated in hospital was in line with the 37.2% reported by Lakshmipriya [81]. Our results of 97.6% awareness were higher than the 85.6% reported among subjects from 5 districts in Ghana, perhaps owing to differences in settings and methodology [82]. The 21% (6/29) lack of awareness of available treatment found in this study was lower than the 30% (33/109) awareness reported in East Nusa Tenggera in Indonesia [83], principally owing to differences in methodology as their study was hospital-based. The 93% awareness rate found in this study was only slightly lower than the 99.7% reported among a cross-section of 767 surveyed subjects in the Lomé neighborhood in Togo [84]. The rate of awareness of 94.9% among subjects in Takeo, Cambodia, was in accordance with the 93% awareness rate reported in this study [85].

The fact that up to 93% of subjects in our study knew that cataract is a blinding disease did not, however, completely translate into them knowing what cataract is. Their understanding of cataract in their local language could range from any visible vision-related problem to a white scar in the eye, depending on the tribe. This is similar to a study in Brazil in which 79% of subjects perceived a cataract as a scar that gradually covers the eye [86].

We report an 86% (25/29) cost-related barrier in this study as the leading barrier to cataract surgery, which is almost twice that (49.2%, 65/132) reported by Fadamiro and Ajite [87] among subjects in Ekiti State, Nigeria. This study was carried out much earlier and over a longer period (2012-2014). The difference in time, settings, and methodology could have led to this disparity. Cost, as a leading barrier to cataract surgery reported in our study, was concordant to that reported among elders in the Nuwara Eliya District, Sri Lanka [88], as a leading barrier to eye care services. Kumar et al [89] reported that 88.9% of surveyed subjects attending an outpatient unit in Uttar Pradesh, India, complained about the cost of cataract surgery and 59.1% complained about the fear of losing sight. These results were similar to the 86% and 59% rates we found for cost and fear, respectively. Our report about cost as a leading barrier was also in line with the reported results by Tafida and Gilbert [90] in a study among subjects in the Jigawa State of northern Nigeria. A study in the English-speaking region of Cameroon found that cost was reported by 52.9% of participants as the major barrier to the uptake of surgery [9]. This was much lower than the 86% we found, principally because their study was a survey based on a questionnaire. Our results about cost as a leading barrier were not very different from the results (91%) reported among 66 subjects in Ghana [91]. Cost of surgery was also among the 3 main barriers to cataract surgical services uptake reported in Benin [92]. This study found that 86% of subjects reported lack of funds as the main barrier to cataract surgery, similar to the 79% reported among 157 subjects in Kilimanjaro, Tanzania [93].

This study found that 59% (17/29) of subjects had fear as one of the leading barriers to cataract surgery, which was similar to the 55.9% reported by Gilles et al [94] within the same setting. The proportion of those who reported fear (59%) as a major barrier to cataract surgery in our study was more than double of that (24.2%) reported in India [95], which could have been because interviews were limited to patients with cataract as opposed to ours and owing to the level of awareness, which should normally be higher in India. Our results about fear of surgery were almost 5 times the 12.57% (8/58) value reported in a similar study in Ghana [91]. Even though the study also made use of 3 FGDs and personalized interviews, their sample was limited to the operated and blind patients with cataract and the study was conducted more than a decade ahead of this study. A study among patients with cataract and key informants in Andhra Pradesh, India, also reported fear as a major barrier to the uptake of cataract surgery [96]. Fear of surgery was also reported by 9.2% of the 2076 surveyed subjects in rural Myanmar [97], which was far below our reported results probably because they used a closed-ended questionnaire with a much larger sample, and was conducted more than a decade earlier as well.

A study in the predominantly English-speaking Southwest Cameroon found that visually impaired patients seek traditional medicine before ever visiting the hospital [9], which was similar to what we found in this study. A study among 60 patients with cataract (operated and blind) in Kilimanjaro, also reported how subjects first sought traditional medicine prior to accepting cataract surgery [98].

In addition to perceived barriers, this study also found that awareness about cataract as a disease that can be cured traditionally also presents as a barrier to accepting cataract surgery. The following excerpt demonstrates this:

…. It is Onyang in Etone [famous tribe]. When you have Onyang, they can use traditional medicine, it works with the Beti [famous ethnic group] but sometimes it fails, after that, you can go to the hospital.

Indirect, Subject #2, FG2

This study reported that the benefits of cataract surgery extended beyond those operated on as the community joined them in celebration when they return to the community. A study in Bangladesh and Kenya also reported how the impact of cataract surgery extended beyond those operated upon [99]. The externalities of successful cataract surgery were also reported among 83 caregivers in Vietnam, whose happiness and life satisfaction among others significantly improved [100]. There is little evidence about the community's perceived impact of cataract surgery. Much of the reported evidence about the postcataract surgical experience is centered around quality of life [101-103].

This study found that 79% of interviewed subjects were happy with free cataract surgery if ever offered. This was lower than the 90% reported among 90 subjects in the Kwale District, Kenya [104]. This difference could have occurred because not only was their sample size larger and had a better male to female ratio (40:50), but also their study was limited to operated and blind patients with cataract (visual acuity≤6/18). Per our results, interviewees who admitted being comfortable with free surgery (79%) were fewer than the 95.2% of 152 patients with cataract who expressed the desire for free cataract surgery in Ghana [105]. The perception of free cataract surgery in some rural communities in Cameroon only compounds the already existing perception of dying on the operating table as reported by Rotchford et al [106]. A high proportion of subjects in this study reported that free cataract surgery was suspected in the community. This was similar to the views expressed among interviewed subjects in Kilimanjaro, suggesting that providing free cataract surgery may not necessarily increase uptake [93].

Our results revealed that fear was the most prominent reason why people outrightly refused cataract surgery. This was concordant with the results reported in Kwale District [107]. Although we did not explore further to find out what constituted subjects’ fear, there is evidence in our results (according to Subject 8 in FGD2, for example) that this could be due to reported poor outcomes of previous surgeries from other clinics.

A study with a much larger sample in Ghana (n=152) also found fear to be the major reason for refusing free surgery [105], similar to our findings. Fear has also been reported to be a major predictor of free cataract surgery refusal in Kenya, principally stemming from rumors [107]. Findings from 46 patients who refused surgery in Paraguay showed that refusal was mainly associated with transport cost and distance from the clinic [108] as opposed to the findings of this study. Their study was based on telephone interviews following a rapid assessment of avoidable blindness survey. A hospital-based study within the same setting also found fear (55.9%) to be the leading reason for refusing free cataract surgery [94]. Fear was also reported among 41 subjects as one of the 3 leading reasons for not taking up eye surgery in rural Eswatini [109].

Refusal of cataract surgery in some communities was more associated with supernatural beliefs, as shown in the following example portraits:

Subject P: He is an operated cataract patient aged 81, widower, and living with the daughter. There is a feeling of belief in superstition when he talks. His expressions relate to experiences of those living with cataracts and the operated *“You already know, in, Africans like promising evil to their brothers […], all that we have as sickness in the hospital we Africans have transformed overnight, that means one can decide to throw the sickness on you like SIDA like cataract then you find yourself with the disease whereby the scientists, big doctors will find it difficult […], while the person responsible will also be aggravating the problem”.*

Subject M: The explanation of their experience portrays the joy that those operated bring to the community but also the lack of money, fear of surgery, age, and transport cost as potential barriers, *“Others are happy, those who do not have money admire those who had surgery. Others say they cannot take surgery because they are old, their small veins will be cut and they will go blind. Others complain of distance, that it is far and that the cost is high going to Obak [MICEI’s location]”.*

### Strengths and Limitations

This is the first ethnographic study in Cameroon, which was aimed at uncovering the challenges faced by community-diagnosed patients with cataract in accessing cataract surgery. As opposed to assumed conditions in other study designs [110,111], the holistic and naturalistic approach of focused ethnography helped to collect detailed qualitative field data that can readily be integrated into practice [46]. The use of both FGDs and personalized interviews led to internal validity. Placing patients, their families, and communities at the forefront of this study was vital for patient-based eye care delivery [112].

This study has 3 main limitations. The study was limited to the Lékié Division, and perhaps the results could have presented more diverse opinions if the focus groups were drawn from different divisions. The sample’s male-female ratio was 1.9:1, which could have led to most of the responses and opinions being skewed toward male subjects. The inaccessibility of some sites reduced the ethnographic diversity.

### Conclusions

This study aimed to explore the challenges in accessing cataract surgery among community-diagnosed patients with cataract and the wider community. We found that cost (25/29, 86%) and fear (17/29, 59%) were the main barriers to cataract surgery compounded by a strong belief in traditional medicine and superstition. These results apply to settings (1) reliant on hospital-based delivery models (2) with a disintegrated eye care delivery from the public health strategy and (3) with little or no health coverage.

This study highlights the overriding need to integrate eye care into the public health strategy and rethink the primary eye care conundrum in Cameroon. Despite evidence in the capacitation of the eye health workforce, the current evidence regarding the integration of eye care into Cameroon’s primary health care is very limited [113]. The current need for renewed knowledge regarding barriers to cataract surgical uptake is indispensable in defining the priorities for primary eye care delivery in Cameroon. Our results reveal that the patient-reported barriers to cataract surgery of those attending eye clinics may not necessarily be the experience reflecting the communities they come from.

This study also shows that because the opinions of indirect subjects represent a major influence over the decisions of the direct subjects to accepting cataract surgery among older people, the decision mechanism is complex as this appears to be a social construct [98].

The following recommendations would therefore be useful: (1) implement a tiered pricing policy and reduce the number of postsurgical visits, (2) consider traditional doctors or healers as major stakeholders and include them in the community health volunteer training program at the clinic, (3) develop a plan for the engagement of mass media for regular awareness raising, (4) train patient counselors and improve cataract surgical outcomes to manage fear, (5) develop and implement advocacy programs including regular community eye talks and aligning eye care delivery with government-led community programs, (6) implement a fee-for-referral service for trained key informants, front line health workers, and traditional doctors, (7) acquire a 4×4 vehicle dedicated to outreach and motorbikes for camp organizers, and (8) deploy ArcGIS and related applications to improve the planning of awareness.

## Appendix

Multimedia Appendix 1 Focus group discussion and interview guide.

## Abbreviations

FGD: focus group discussion
ICO: International Council of Ophthalmology
IRB: institutional review board
MICEI: Magrabi ICO Cameroon Eye Institute
PI: principal investigator
SiB: Seeing Is Believing

## Data Availability

Data will be made available upon request.
