# Supplementary material for: Exploring the Reasons for Low Cataract Surgery Uptake Among Patients Detected in a Community Outreach Program in Cameroon: Focused Ethnographic Mixed Methods Study
Source: JMIRx Med. 2022 Jun 9;3(2):e35044. doi: 10.2196/35044 (PMC10414320; doi:10.2196/35044)
Supplement: Multimedia Appendix 1 [file xmed_v3i2e35044_app1.docx]

## Exploring The Reasons for The Low Cataract Surgical Uptake Among Patients Detected in a Community Outreach Program in Cameroon: Focused Ethnographic Mixed-Method Study

**FGD and Interview Guide**

**Study Objectives:**

1. Understand people’s knowledge about cataract and available treatment (Theme 1)
2. Find out patients’ barriers to the uptake of cataract surgical services (Theme 2)
3. Understand people’s perception about the outcome of cataract surgery (Theme 3)
4. Find out people’s perception about free and paid cataract surgery (Theme 4)
5. Explore the reasons for the unwillingness to take cataract surgery (Theme 5)

**QUESTIONS**

**CODE 1: What do you know about cataract?**

Probes

- How is it called in your community and what do people think about it?
- Do you think it can be treated and how?

**CODE 2: What would you say hinders people who want cataract surgery from taking cataract surgery?**

Probes:

- Suppose someone wants surgery, what may disturb him/her from finally taking surgery?
- Do they go to other places for cataract surgery? If so where and why?

**Code 3: What do people say when those who had cataract surgery come back to the community?**

Probes:

- Are they happy with the surgery? If not why?
- Do people usually complain and about what?

**Code 4: What is your opinion about free and paid cataract surgery?**

Probes:

- How do people consider free cataract surgery in your community?
- How do people consider paid cataract surgery in your community?

**Code 5: Why do you think some people do not want cataract surgery?**

Probes:

- Are there any reasons that may make someone not want to take cataract surgery?
- What can be done to encourage people to take up cataract surgery?
